# Modulation of Skin Cancer by the Stimulator of Interferon Genes

**DOI:** 10.3390/genes14091794

**Published:** 2023-09-13

**Authors:** Max Oscherwitz, Victoria Jiminez, Hanna Terhaar, Nabiha Yusuf

**Affiliations:** 1 Heersink School of Medicine, University of Alabama, Birmingham, AL 35294, USA; 2 Department of Dermatology, University of Alabama, Birmingham, AL 35294, USA

**Keywords:** STING, interferons, skin cancer, melanoma, basal cell carcinoma, BCC, squamous cell carcinoma, SCC, immunotherapy

## Abstract

Morbidity and mortality from skin cancer continue to rise domestically and globally, and melanoma and non-melanoma skin cancers are a topic of interest in the dermatology and oncology communities. In this review, we summarize the stimulator of interferon genes (STING) pathway, its specific role in the pathogenesis of DNA damage and skin cancer, and STING-specific therapies that may fight both melanoma and non-melanoma skin (NMSC) cancers. Furthermore, we discuss specific portions of the STING pathway that may be used in addition to previously used therapies to provide a synergistic effect in future oncology treatments and discuss the limitations of current STING-based therapies.

## 1. Introduction

The multi-factorial burden of skin cancer continues to rise rapidly on both a domestic and international scale. Roughly 20 percent of United States citizens will have some form of skin cancer by age 70, and it is predicted that by the year 2030, the United States will have over 112,000 new cases of melanoma [1,2,3]. This poses a challenge to healthcare providers in all fields, but particularly those in the field of geriatric medicine. From an economic standpoint, the country spends almost USD 8.1 billion on skin cancer-related costs annually [4]. Efforts in both prevention and intervention of skin cancer are crucial to improve morbidity and mortality and mitigate the financial impact of skin cancer on individuals and communities.

Although multiple types of skin cancers exist, they can broadly be classified into two major categories: non-melanoma skin cancer (NMSC) and melanoma cancer (MC). Examples of non-melanoma skin cancer include basal cell (BCC) and squamous cell (SCC) carcinomas. NMSC has an estimated incidence of 18–20 times higher than melanoma; BCC and SCC make up 99% of NMSCs [5,6]. Although melanoma is responsible for only 1% of all malignant tumors within the body, its aggressive nature makes it quite lethal, with specific subtypes boasting 5-year survival rates of only 15–20% [6].

Recent advances in immunotherapy have brought forth new signaling pathways that are hypothesized to be directly linked to the pathogenesis of skin cancer. These have the potential to revolutionize oncological treatments of skin cancers and are gaining traction in both the dermatology and oncology communities. The stimulator of interferon genes (STING) pathway is a promising anti-tumor adapter protein with the potential to oppose tumor growth [7,8]. Several studies have shown that STING may be particularly efficacious in improving the efficacy of chemotherapy in the fight against both NMSC and MC [7]. Additionally, the pathway has also been investigated in the pathogenesis of Merkel cell carcinoma and adult T-cell leukemia and lymphoma [7]. Specifically, existing research has emphasized the importance of type 1 interferon (IFN) on the ability of the STING pathway to augment second messengers, undergo epigenetic modifications, and the connection with STING and other autoimmune processes [7,8].

More in-depth studies are required to fully understand how STING therapy may be best utilized in both the oncology and dermatology fields. Furthermore, novel therapies utilizing STING technology may have the ability to target solid tumors within multiple organ systems. This review will provide a greater understanding of how STING works on a molecular signaling level and a summary as to how it may be used in future oncologic therapies.

## 2. Epidemiology and Brief Classification of Skin Cancers

Extensive exposure to sunlight and ultraviolet (UV) radiation is the most important external risk factor for developing malignancy involving the skin [9]. UV radiation is both a carcinogen and DNA-damaging agent that has been demonstrated to promote tumor growth [9]. Specifically, UVA and UVB rays from the earth’s atmosphere can cause damage to keratinocyte DNA, and UVB tends to have a more significant carcinogenic effect than UVA [10]. UVA and UVB are hypothesized to induce oxidative stress, upregulate teratogenic proteins, and recruit inflammatory factors within epidermal cells to induce DNA damage and lead to uncontrolled replication [10,11,12].

Normal human skin comprises both the superficial epidermis and the deep dermis, as well as a neighboring hypodermis; these layers serve as part of the innate immune system to aid in host defense. The epidermis has five layers: the stratum corneum (most superficial), stratum lucidum, stratum granulosum, stratum spinosum, and stratum basale (most deep). Specialized cells make up the epidermis, including melanocytes, keratinocytes, Merkel cells, and Langerhans cells; each of these has a distinct role vital for the normal mechanical and immune function of the skin [11]. When describing skin cancer, the main difference between NMSC and MC lies in their cells of origin. Basal cell carcinoma arises from the stratum basale layer of the epidermis, while squamous cell cancer arises from keratinocytes within the outermost layers of the epidermis [11]. Melanoma originates from mutated melanocytes, also found within the stratum basale layer of the epidermis. Taken together, it is crucial to understand the anatomy of human skin in order to gain a comprehensive understanding of the pathogenesis of skin cancer.

The detection of skin cancer is multi-factorial and includes both patients and healthcare providers. Individual cancers may be detected macroscopically or microscopically, and annual skin checks are crucial in identifying early-stage skin cancers before they can metastasize. In terms of simplicity, skin checks can be conducted without the need for extensive equipment apart from a dermatoscope. These examinations are efficient as well, taking only minutes out of a patient’s daily schedule. To ensure comprehensive care, clinicians must obtain a detailed clinical history, perform thorough physical exams and self-examinations, and ensure the patient’s adherence to future appointments.

In terms of prevalence, BCC is the most observed subtype of skin cancer overall. These malignancies have a low metastasis rate and have an age-adjusted mortality rate of 0.12 per 100,000 person-years [13]. BCCs are often located on the face and can be identified by their characteristic pearly appearance with rolled borders on gross examination. Additionally, they often are found as papules with underlying telangiectasias under dermoscopy. Treatment options for BCC are primarily based upon excisional surgery, curettage, and topical therapy and are highly efficacious, with a 5-year survival rate close to 100% when appropriately treated, per the Canadian Cancer Society [14]. 

Conversely, SCC is the second most observed subtype of NMSC. Compared to BCC, these have a higher rate of metastasis, which occurs at a rate of 3–9%, as shown by previous studies [15]. The age-adjusted mortality rate for SCC is still relatively low, recently being reported at 1 per 100,000 person-years [16]. Though SCC can vary in appearance, a typical lesion is composed of scaly, thick, or erythematous crusted skin, which in some cases may resemble a poorly healed sore. Treatment options are like those of BCC, with survival rates close to 99% [17].

Although less common than NMSC, melanomas are one of the deadliest skin malignancies. These cancers comprise 1% of total cancers but account for 80% of total deaths [18]. Melanomas vary in phenotypic appearance but share many features with benign pigmented nevi. These cancers may exhibit asymmetry, irregular borders, variegation in color, and may increase in size quite rapidly. Treatment options for melanoma are diverse and are rapidly advancing. These include surgeries, targeted immunotherapies, radiation, and chemotherapy. In total, 5-year relative survival rates depend on staging, spread, and melanoma type, and, according to the American Cancer Society, can range from over 99% if localized to 32% if distantly metastasized [19]. Advocacy efforts are gaining traction within the dermatology community to increase public awareness of the dangers of untreated melanoma.

## 3. What Is STING (Stimulator of Interferon-Related Genes)?

New technological capabilities have brought the STING signaling pathway to the forefront of the oncology world. The pathway was first described in 2008 as a eukaryotic defense mechanism against viruses [20]. Since this time, the pathway has gained notoriety for its activity against different types of cancer and its ability to potentiate oncologic therapies. At a fundamental level, the pathway is an inflammatory response to double-stranded DNA [20]. Specifically, STING upregulates type 1 IFN production [7,20].

The human immune system’s normal response to foreign antigens is accomplished by two distinct parts of the immune system. Physical barriers, including skin and mucosal membranes, comprise the innate immune system, providing physical and molecular protection defenses against invading organisms and molecules. Conversely, the adaptive immune system comprises cells with specialized functions that interact in order to produce antibodies, activate complement, and activate memory responses in order to identify and eliminate pathogens. Antigens from foreign molecules are ingested and processed by antigen-processing cells, which present these molecules to specialized T-cells via major histocompatibility complexes. Once presented, the antigens can then go on to activate other cells, molecular cascades, signaling pathways, and cytotoxic host responses. 

The immune system produces various inflammatory cytokines and IFNS in response to acute pathogenic invasion. Of note, there are currently three major IFN classes. Although each family exhibits differences in potency, receptor type, and specific downstream messengers, the STING pathway is most closely intertwined with type 1 IFNs.

Type 1 IFNs allow host cells to defend themselves against foreign viruses, bacteria, and fungi and can activate other downstream inflammatory pathways [21]. This class has also been hypothesized to exhibit anti-malignancy properties; recent studies have focused on identifying a stimulus to promote a Type 1 IFN response. An intracellular receptor resides within the endoplasmic reticulum and activates type 1 IFNs via a well-documented cGAS-STING pathway [22]. Importantly, type 1 IFNs have also been implicated in the pathogenesis of other diseases, including pulmonary disease [21].

If non-native double-stranded DNA is detected within the cytoplasm of a host cell, the cGAS-STING pathway is initiated [23]. Once the foreign material encounters cGAS, a sensing protein, a conformational change occurs, facilitating the formation of a molecule of 2′,3′-cyclic GMP-AMP. Additionally, this step leads to an interaction between GTP and ATP, which ultimately results in the activation of STING, which is housed within the endoplasmic reticulum when inactive [23,24]. Simultaneously, palmitoylation of TANK-binding kinase 1 occurs, which leads to the recruitment of IFN regulatory factor 3 and the phosphorylation of STING [23,24]. This sequence is hypothesized to cause a conformational change, allowing STING to translate through the Golgi apparatus and move towards the perinuclear region via the assistance of several modulators, including iRhom2 [23,24]. The final event in this sequence is the translocation of IFN regulatory factor 3, which travels to the nucleus. Once it reaches its destination, the transcription of Type 1 IFN genes is stimulated. 

## 4. Role of STING in DNA Damage

The previously mentioned innate immune system reacts to foreign nucleic acids via RNA and DNA sensing receptors to mount a host immune response [25]. DNA infiltration into cells can propagate various changes, including senescence, replication, mitochondrial stress, and others that lead to the up-regulation of type I IFN [25]. Activation of pattern recognition receptors (PRRs), specifically the cGAS/STING pathway, is activated when the presence of double-stranded DNA (dsDNA) of foreign invaders is detected [25]. The identification of dsDNA as a signaling mechanism for STING activation has prompted interest in its ability to be a therapeutic target against carcinogenesis. Downstream of the STING signaling pathway lies its effects on host cell DNA [25]. Additionally, the pathway can be triggered if nuclear or mitochondrial dsDNA inappropriately leaks into the cytosol of cells. Mis-segmented chromosomes after mitosis can become dislodged from their original position within chromatin, obtain a nuclear envelope, and form micronuclei, a hallmark of genomic instability [25]. These encapsulated micronuclei are subject to irreversible membrane rupture, escape into the cytosol, and activate cGAS-STING [25,26]. This is frequently identified in tumors, immortalized epithelial cells, and fibroblasts. The fact that STING signaling is present throughout mitosis allows for targeting this pathway via cell cycle regulation in rapidly dividing cancer cells [25].

Mitochondrial DNA damage also activates this pathway, and degradation has been shown in malignancies to trigger cGAS-STING. Cytosolic mitochondrial DNA release acts as a ligand for pathway potentiation, causing IFN-mediated cell death that may be a target for future therapies [25,27]. This immunologic role of cGAS and STING has been demonstrated to be diminished in lung adenocarcinoma, late-stage gastric cancer, and invasive breast ductal carcinoma, leading to poor survival [25,28,29,30].

Cancers evade the cGAS-STING-mediated signaling once DNA is sensed via targeting gene expression or suppressing their function. Suppression of cGAS-STING signaling downregulates apoptotic and senescence pathways, increasing the protection of malignant cells from host tumor surveillance [25,31]. Under hypoxic conditions, cGAS-STING signaling decreases simultaneously with the release of mitochondrial DNA, both leading to muted anti-tumor responses, further evidencing the necessary role of STING signaling for anti-tumorigenesis [25,28,32]. Increased host vulnerability in these states also leaves host cells more susceptible to oncogenic viral replication, such as in the herpes simplex virus.

## 5. Role of STING in Immunotherapy of Skin Cancer 

Given the exhibited anti-oncogenic properties of STING, it is no surprise that new advances in oncology have investigated this pathway to target skin cancer tumorigenesis. Several studies have explored how STING responds to damaged DNA released from lysed tumor cells [33]. In addition to decreased DNA repair mechanisms, tumor cells exhibit replicative immortality, incorporating the nucleoside analog 6-thio-dG into the growing telomeres [34]. This nucleoside can become imbalanced, also resulting in damaged DNA that can be detected by the normal cells in the body [34]. This leads to the activation of IFNa and IFNb, which ultimately increases the presentation of oncogenic antigens to the recruited CD8+ T-cells while promoting additional cell apoptosis through NK cells [33]. By understanding that the STING pathway is driven by damaged dsDNA, therapies for skin cancer may be able to target the accessibility and recognition of the dsDNA by antigen-presenting cells. 

### 5.1. Melanoma

Although checkpoint inhibitor therapies currently exist to target skin cancer, their primary mechanisms do not primarily rely on inducing damage to dsDNA in tumor cells. Therapies for malignant skin cancer, such as radiotherapy and chemotherapy, rely on the STING pathway for regulating type 1 IFN production. Other documented mechanisms such as inducing DNA damage and forming micronuclei, inducing apoptosis, and exposing the damaged DNA to PPRs on the cell surface have been described [35]. Tumor cells have been found to suppress STING’s activity, resulting in resistance to these treatments [36,37].

Drugs that target, the DNA repair enzymes or replication interfere with producing dsDNA recognizable to the host cell, thus activating the STING pathway [38,39]. Topoisomerase inhibitors have been found to promote antigen expression in multiple melanoma lineages and T-cell induction of IFNs due to their ability to promote abnormal DNA replication [40]. Damaged dsDNAs, produced by drugs that impede DNA repairs and replication, ultimately stimulate the STING pathway and, therefore, can be seen as potential therapies in the induction of the STING pathway in the fight against skin cancer. 

STING agonists are another type of immunotherapy currently being studied for their efficacy on the tumor microenvironment of the integumentary system. In one study, when STING agonists were injected intratumorally into subcutaneous melanomas, the pathway increased the production of local anti-angiogenic factors, chemokines, and LTbR agonists, ultimately aiding in the restoration of the normal vasculature and promotion of local tertiary lymphoid structures resulting in the slow growth of the tumor microenvironment [41]. Although the external validity of this study was limited to murine species, human STING agonists that target skin tumors are currently being developed, and have exhibited positive results in clinical trials [42]. However, further development of these agonists is needed in order to increase their responsiveness and manipulate the mechanism of agonist entry into the cells due to underlying hydrophilicity [33].

Recent studies exploring injection techniques in intertumoral drug delivery, drug formulation, and tumor stroma on the efficacy of the STING agonist on tumor cells have found that STING agonists are significantly more effective in soft tumors such as melanoma than firm tumors such as colorectal cancer [43]. Additionally, multisided-hole needle delivery led to an increased IFN response compared with end-hole sting delivery, and agonists encapsulated in a hydrogel were found to have a greater intertumoral permeation [43]. These findings are essential when considering vehicles of administration. 

STING agonists, combined with drugs that target downstream mechanisms within the STING pathway, have been found to induce significant anti-tumor responses [44]. Research has found that stimulating the STING pathway, and inhibiting epigenetic modifiers that silence the STING pathway, has shown success in inhibiting tumor growth in patients that have not been responsive to previous types of immunotherapy [45].

### 5.2. Squamous Cell Carcinoma

There are limited non-surgical options for treating SCCs, with radiation therapy being a popular treatment for this cancer [46]. Similar to melanoma, CTLA-4/PD-1 inhibitors are the primary immunotherapy treatment. STING has been found to stimulate the PD-1 pathway. Therefore, STING therapy with CTLA-4/PD-1 inhibitors may further increase the efficacy of prohibiting cancerous growth [7]. Low expression of STING has also been found to be associated with worse outcomes in squamous cell carcinomas located on the head and neck regions [47]. In addition to the pathway’s main anti-tumor effects, the same study found that an alternative pathogenesis associated with the STING pathway manipulates ROS homeostasis to induce DNA damage in the same patients [47]. Due to these findings, research should further aim to develop new therapies that aim to stimulate the STING pathway for the treatment of SCCs. 

### 5.3. Basal Cell Carcinoma

Like SCC, radiation and topical chemotherapy are amongst the more popular non-surgical options for BCC. There are currently no FDA immunotherapy approvals for advanced or metastatic BCCs [48]. Due to the main etiological factor for BCC being UV exposure, this NMSC is a prime target for the STING pathway due to the dsDNA damage precipitated by sun exposure. Research has found that IFNa treatment induces BCC regression by inducing suicide through CD95 receptor–CD95 ligand interaction [49]. These findings emphasize the need for future therapy development manipulating the STING pathway as STING pathogenesis includes activation of IFNs such as IFN-a.

### 5.4. STING Resistance in Tumor Cells

STING is vital in prohibiting tumor growth, but to adapt to this, tumor cells have evolved to inhibit aspects of the STING pathway. Research has found that CD8+ T-cells in cancer patients have decreased expression of the STING pathway and are less successful in promoting an anti-oncogenic response. Therefore, when the pathway in CD8+ T-cells that have decreased expression of the pathway was stimulated, the cells were more successful in promoting an anti-tumor response by increasing differentiation of stem-cell CD8+ cells [34]. Tumor cells have also targeted epigenetic modifications and degradation of the STING pathway to suppress its anti-oncogenic effects [50,51]. Research has found that loss of STING function prohibited melanoma cells from producing type 1 IFNs after exposure to damaged dsDNA [49].

## 6. Further Potential Therapies

Further potential therapies include the injection of mRNA-lipid nanoparticles of constitutively active STING mutants into the cancer cells, which have been found to reactivate STING anti-tumor immunity and promote apoptosis of tumor cells [52]. This mechanism of action does not induce anti-proliferative effects in lymphocytic cells that could result in cytotoxicity as seen with STING agonists and proves to be a potential therapy to treat “cold tumors”. Potential therapies that have yet to be explored include targeting cytosolic DNA sensors such as AIM2 that have an antagonistic effect on the STING pathway [53]. Further research with STING agonist combination therapy includes combining the agonist with a protein-based cancer vaccine [54]. Positive results were observed as this promising therapy caused IFN and TNFa production levels, promoting CD8+ and CD4+ T-cell infiltration and function while polarizing CD4 T-cells towards TH1 differentiation [54]. Additionally, STING combined with a cancer vaccine was shown to decrease the presence of immune-suppressive cells surrounding the tumor; however, most of the oncogenic cells escaped from immune surveillance [54]. Therefore, further modification of this therapy is required in order to increase its efficacy and minimize tumor escape. The role of cGAS-STING activation, when paired with existing cancer treatments, poses beneficial potential in maximizing responses. The synergistic effects of these vaccines, if combined with chemotherapy or radiotherapy, and immune checkpoint blockade (ICB) therapy are of particular interest. Recent work has suggested that radiation therapy enhances anti-tumorigenesis via immune activation by cGAS-STING. Resistance mechanisms against this have been demonstrated [25,55,56,57,58]. However, it has been proposed that combinations of radiotherapy with cyclic dinucleotides (CDNs) like c-di-GMP and cGAMP acting as downstream second messengers for STING activation might enhance tumor clearance [25]. Where it has been shown that CDN monotherapy can suppress tumor growth via innate immune signaling, combining with radiotherapy allows for CD8+ T-cell involvement and synergy [25,59]. Vaccine combinations with irradiated tumor cells that display GM-CSF and CDNs have also exhibited anti-oncogenic responses in melanoma, SCC, and other non-cutaneous malignancies [25,60]. Regarding immune checkpoint inhibitors, it has been observed that STING-deficient mice respond poorly in comparison to wild-type mice undergoing anti-CTLA4 and anti-PDL1 immunotherapies [25,61,62,63,64]. Thus, CDN-induced STING signaling combined with ICB therapies also enhanced the CD8+ T-cell response and anti-tumor attenuation [65].

## 7. STING and Clinical Trials

To date, several clinical trials have been initiated for treating head and neck squamous cell carcinoma (HNSCC) with STING agonists including ADU-S100, SB-11285, and MK-1454 [66,67,68]. These have been combined with various co-therapies including pembrolizumab and atezolizumab, and thus far, limited data has been obtained (Table 1) [66,67,68].

In the Phase 2 clinical trial that utilized ADU-S100 (a STING-agonist that functions as a synthetic cyclic dinucleotide) co-administered to patients with PD-L1 positive recurrent or metastatic HNSCC, the endpoints of the study were patient safety, preliminary anti-tumor activity, pharmacokinetics, and immunomodulation [66,69]. Sixteen non-Hispanic or Latino participants were recruited, and all participants received at least one dose of the study drug [66]. Six out of the sixteen (37.5%) participants died during the study, and sixteen out of the sixteen participants (100%) experienced at least one serious or non-serious adverse event [66,69]. Fatigue was the most common adverse event experienced by nine out of the sixteen (56.25%) participants, while diarrhea, nausea, and weight loss were the second most common adverse event, each experienced by eight out of the sixteen (50%) participants [66,69]. Overall, one participant experienced a complete response to the treatment, while five participants experienced a partial response [66,69].

## 8. Conclusions and Perspectives

**Figure 1:**
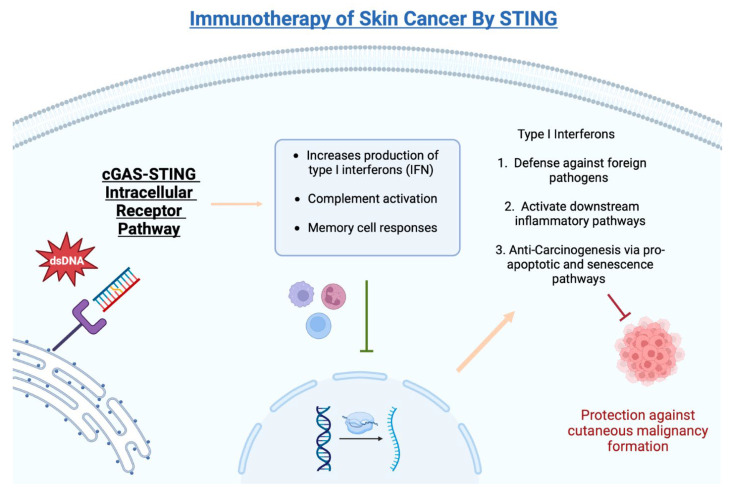
Biological functions of cGAS-STING intracellular pathway.

The cGAS-STING signaling pathway has gained significant attention regarding its anti-tumor properties in certain malignancies, especially skin cancer. As demonstrated in Figure 1, STING plays a vital role in the fight against foreign pathogens, the activation of downstream inflammatory pathways, and anti-carcinogenesis via pro-apoptotic and senescence pathways. Put simply, the STING pathway functions by detecting the presence of DNA, which subsequently leads to the activation of inflammatory modulators for host defense, anti-inflammatory, and anti-oncogenic effects.

STING’s effects on immune modulation pose benefits in both monotherapies and heightened efficacy in combination with other existing forms of both NMSC and melanoma skin cancers. While this pathway has been extensively studied in vitro, further animal and human studies and trials are needed to investigate further the safety and reliability of cutaneous cancer treatment optimization. Furthermore, although clinical trials are currently underway, more data and trials are needed to draw definitive conclusions about the efficacy of the STING pathway in oncology therapy. Collaboration between researchers and clinicians within the oncology and dermatology fields is necessary to push forward the next generation of STING treatments. 

## Figures and Tables

**Table 1:** STING agonist clinical trials for squamous cell carcinomas.

Agonist	Indications	Recruitment Status	Phase	Route	Co-Therapy	Results	NCT Number
ADU-S100	PD-L1 positive recurrent or metastatic HNSCC	Terminated	II	I.T.	I.V. infusions of pembrolizumab	No significant anti-tumor response was observed	NCT03937141
SB 11285	Melanoma, HNSCC, and advanced solid tumors	Active	I	I.V.	Dose-escalation study: administered as a monotherapy, then combination with atezolizumab	N/A	NCT04096638
MK-1454	PD-L1 positive recurrent or metastatic HNSCC	Completed	II	I.T.	I.V. infusions of pembrolizumab	No study results posted	NCT04220866

Abbreviations: HNSCC, head and neck squamous cell carcinoma; I.V., intravenously; I.T., intratumorally.
